# Elongation of the Aorta after Thoracic Endovascular Aortic Repair: A longitudinal study

**DOI:** 10.3390/ijerph17041205

**Published:** 2020-02-13

**Authors:** Chun-Ku Chen, Hsiao-Ping Chou, Ying-Yueh Chang, Chun-Che Shih

**Affiliations:** 1 Department of Radiology, Taipei Veterans General Hospital, Taipei 11217, Taiwan; hpchou@vghtpe.gov.tw (H.-P.C.); yyjhang@vghtpe.gov.tw (Y.-Y.C.); 2 Department of Radiology, Faculty of Medicine, School of Medicine, National Yang-Ming University, Taipei 11221, Taiwan; 3 Division of Radiology, Yonghe Cardinal Tien Hospital, New Taipei City 23445, Taiwan; profccshih@gmail.com; 4 Institute of Clinical Medicine, School of Medicine, National Yang-Ming University, Taipei 11221, Taiwan; 5 Division of Cardiovascular Surgery, Taipei Municipal Wanfang Hospital, Taipei Heart Institute, Taipei Medical University, Taipei 11696, Taiwan

**Keywords:** aorta, elongation, thoracic endovascular aortic repair

## Abstract

Aortic morphology is associated with age, with the diameter being larger in older people. Thoracic endovascular aortic repair (TEVAR) is a treatment for aortic diseases, such as aortic dissection. When evaluating patients, aortic elongation could interfere with the classification of TEVAR complications. The longitudinal change in aortic length has not been studied in detail. In patients receiving thoracic endovascular aortic repair between 2007 and 2013, we determined the aortic length between the sinotubular junction, left common carotid artery, subclavian artery, and celiac artery on their first five annual follow-up computed tomography (CT) exams. Using the immediate post-TEVAR follow-up CT as the comparison reference and a lengthening of the aortic segment by 10 mm or more as the definition of elongation, 16 of 41 (39%) showed elongation between the innominate artery and celiac artery. When compared with the immediate follow-up CT, a higher proportion of patients showed elongation at the fifth year’s follow-up CT than the first year’s follow-up CT (*p* < 0.01), and the average lengthening per year was 1.7 mm. There was progressive lengthening of the aorta after TEVAR.

## 1. Introduction

Aortic morphology has been reported to be associated with age. A study of 123 subjects without thoracic aorta pathology or surgery found that the aortic diameter enlarges, the posterior arch elongates, the tortuosity index decreases, and the attachment zone angle is larger in older people [1]. Age had a correlation coefficient of 0.61 with arch length (*p* < 0.01) in the study. Another study consisted of 210 consecutive patients who received a computed tomography (CT) scan and found that the thoracic aorta was significantly related to age, with a correlation coefficient of 0.54 [2]. Patients with type A aortic dissection have a longer ascending aorta than do healthy people [3]. Ascending aortic length per centimeter was reported to have an odds ratio of 5.3 for ascending aortic dissection [4]. However, these studies were cross-sectional studies, and the exact longitudinal change of aortic length was not clear.

Thoracic endovascular repair (TEVAR) is increasingly utilized as the treatment of choice in treating aortic pathology such as aortic dissection, blunt thoracic aortic injury, and rupture aneurysms. A study found that, from 2007 to 2015, the blunt thoracic aortic injury patients receiving TEVAR increased from 12.1% to 25.7%, while those receiving open repair decreased from 7.4% to 1.9% [5]. TEVAR is less invasive than open repair and a study showed that endovascular treatment has the advantages of shorter in-hospital stays and less in-hospital mortality and has a comparable functional status for treating ruptured descending thoracic aortic aneurysm [6]; however, patients who receive TEVAR could have stent graft-related complications, including endoleak [7], stent graft migration, and stent graft collapse [8]. Reintervention was performed in 11.5% of type B dissections after TEVAR, for conditions such as stent graft-related complication [9]. While the rate of migration has been reported to be 4% [10], aortic elongation could interfere with the process of deciding whether there was migration of stent graft [11], and elongation could be mis-interpreted as stent graft migration [12]. 

The method for migration estimation proposed previously has stated that the aortic length should be stable before using a landmarked base measuring the distance between the stent graft and the anatomical landmark, for example, the distance along centerline of the aorta, between the left subclavian artery and celiac artery, should be stable before measuring and interpreting whether the stent has migrated or not [13]; a study has calculated the rate of migration using the above criteria, however, the duration between the follow-up CT and the operation was not clear [11]. It is not unclear how the aortic length would change in individuals in a long-term longitudinal follow-up study. In this current study, we analyze a cohort of patients receiving TEVAR with regular follow-up and analyze the change in the aortic length.

## 2. Materials and Methods 

### 2.1. Subjects

Patients receiving TEVAR from 2007 to 2013 were selected. Patients without immediate follow-up CT were excluded, patients who did not have follow-up in each of the five years following TEVAR were excluded, patients who had reintervention in the aorta were excluded, and patients who had stent graft-related complications, such as stent graft-induced new entry and stent graft kinking were excluded. The age, gender, comorbidities (including hypertension, previous coronary artery disease, chronic obstructive pulmonary disease, diabetes mellitus, chronic renal failure, and hyperlipidemia) were recorded. Procedure characteristics including stent graft number, stent graft type, and stent graft size were also recorded. This retrospective study was approved by the local institutional review board, and the need for informed consent for the study was waived (VGHIRB No.2019-11-005CC). 

### 2.2. CT Scan Parameters

CT was performed by using a 64-detector row scanner (Aquilion 64; Canon Medical Systems, Otawarashi, Japan). Informed consent for the CT scan was obtained. The Bolus tracking method was used, with the region of interest (ROI) placed at the ascending aorta. Iodinated contrast medium with 320–370 mg/mL of iodine was injected at a rate of 3.5 mL per second. After the density at the ROI reached 150 Hounsfield units, the scan was performed with 120 or 100 kVp, depending on the patient size, and the rotation time was set at 0.5 s. Automatic dose modulation was applied with the standard deviation set at 20, and an mA set between 10–500 mA. Images were reconstructed to a 1 mm slice thickness with an interval of 0.8 mm.

### 2.3. TEVAR Procedure

At the time of the TEVAR procedure, the proximal landing zone was selected according to the pre-TEVAR planning with CT images to cover the primary entry site or to achieve adequate seal. The selected stent graft size was approximately 10%–20% larger than the diameter of the native aorta. Whether a tapered or nontapered device was used depended on the difference between the diameter of the proximal and distal landing zones. The stent grafts used were stainless-steel-based stent grafts covered with a polyester graft (Zenith TX2 or TX2 Proform, Cook Medical, Bloomington, IN, USA), or nitinol-based stent grafts (GORE TAG, W.L. Gore & Associates, Inc., Flagstaff, AZ, USA; Valiant, Medtronic PLC, Santa Rosa, CA, USA). The stent graft was deployed into the targeted proximal landing zone under fluoroscopic guidance or the wire. After the TEVAR procedure, follow-up CT scans were performed before the patient was discharged (as immediate post-TEVAR CT hereafter), at 6 months, at one year, and then annually.

### 2.4. Aortic Length Measurement

**Figure 1:**
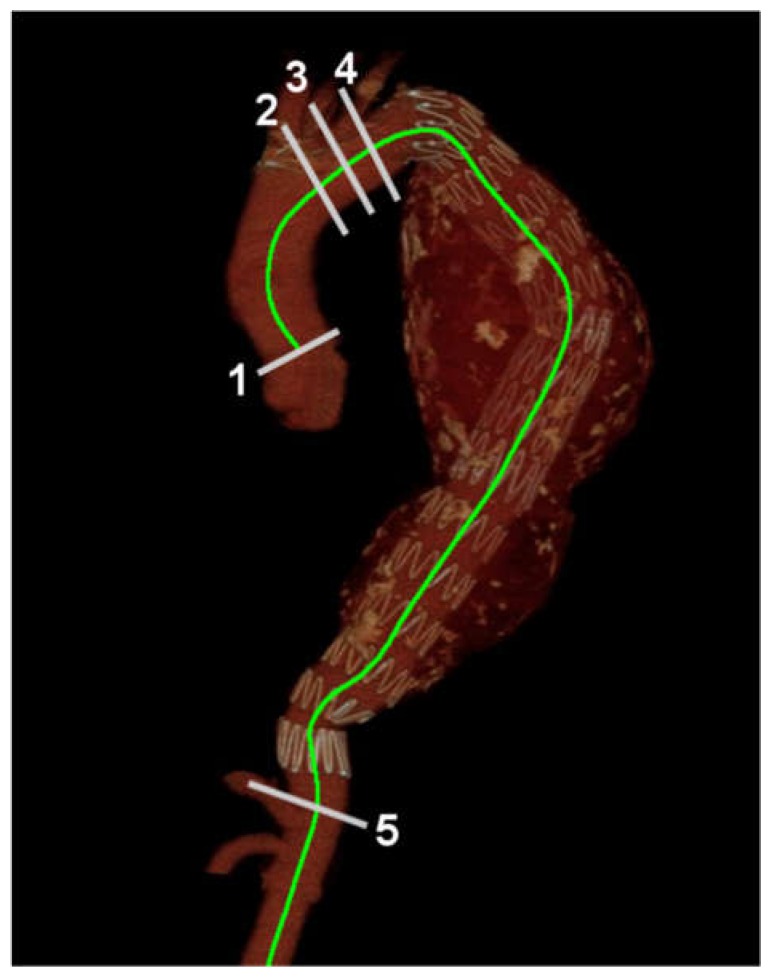
Measuring aortic length. 1, Sinotubular junction (STJ); 2, innominate artery (INA); 3, left common carotid artery (LCCA); 4, left subclavian artery (LSCA); 5, celiac artery (CA). The aortic length along the aortic luminal centerline (green curved line) between the STJ–INA, INA–CA, LCCA–CA, and LSCA–CA were measured.

The CT images were loaded to the workstation (Aquarius iNtuition, Terarecon Inc., Foster City, CA, USA). The centerline was semi-automatically extracted; the sinotubular junction was designated as starting point. The workstation calculated the path length along the centerline. The path length along the centerline between the sinotubular junction (STJ) and the innominate artery (INA), the path length along the centerline between INA and the celiac artery (CA), between the left common carotid artery (LCCA) and CA, and between the left subclavian artery (LSCA) and the CA were calculated for each year’s follow-up CT (Figure 1).

### 2.5. Statistical Analysis

The longitudinal length differences in the INA–CA length as compared with the immediate post-operative CT, at the first, second, third, fourth, and fifth annual follow-up CT, were calculated. The differences regarding other segments, STJ–INA, LCCA–CA, and LSCA–CA, were also calculated. The length difference between the individual annual follow-up CT and the immediate post-TEVAR CT was compared by the Wilcoxon signed-rank test. The growth rate was estimated by the length difference between the fifth year and the immediate post-TEVAR follow-up CT, over the follow-up duration of 5 years. A lengthening of aortic length by 10 mm or more between STJ–INA, INA–CA, LCCA–CA, and LSCA–CA was classified as having elongation; those without lengthening of the aforementioned aortic segments by 10 mm were classified as having no elongation. 

The percentage of patients having elongation was calculated. The patients were then stratified into group A (INA–CA lengthening by less than 10 mm) or group B (INA–CA lengthening of 10 mm or more). Factors including the patient, aorta, and procedure characteristics were compared between group A and group B by the chi-square test or by the Mann–Whitney U test. All analyses were performed by using R statistics (Version 3.6.0, R Foundation, Vienna, Austria). *p* < 0.05 was considered to be statistically significant.

## 3. Results

According to the criteria in the methods section, a total of 41 patients were included; 88% were male, and the median age was 56 years. Eighty-one percent of the patients had hypertension, 10% had diabetes mellitus, and coronary artery disease was noted in 7% of the patients. Fifty-one percent had aortic dissection, and 42% had complications at presentation. About half of the patients had received implantation of more than one stent graft, and 80% had received stainless-steel-based stent grafts. The median diameter of the stent grafts was 36 mm (Interquartile range (IQR), 4 mm) and the median length of the stent grafts was 194 mm (IQR, 48mm). The patient, aortic lesion, and TEVAR procedural characteristics are listed in Table 1.

### 3.1. Aortic Length

The median INA–CA length was 283 mm (IQR, 30 mm) at the immediate post-TEVAR follow-up CT. The median INA–CA length at the first, second, third, fourth, and fifth year follow-up was 285 mm (IQR, 37 mm), 289 mm (IQR, 42 mm), 289 (IQR, 42 mm), 291 (IQR, 41 mm), and 294 mm (IQR, 41 mm) (Table 2), and these were longer than the length at the immediate post-TEVAR CT (*p* < 0.01 for all five years).

The median pre-TEVAR STJ–INA length was 88 mm (IQR, 14mm); the STJ–INA length at the first yearly follow-up CT has a median value of 87 mm. This shows no significant change compared with the immediate post-TEVAR follow-up CT (*p* = 0.45). The STJ–INA length at the second, third, fourth, and fifth year was longer than that at the immediate post-TEVAR follow-up CT (*p* = 0.02, *p* = 0.04, *p* < 0.01, and *p* < 0.01, respectively).

**Figure 2:**
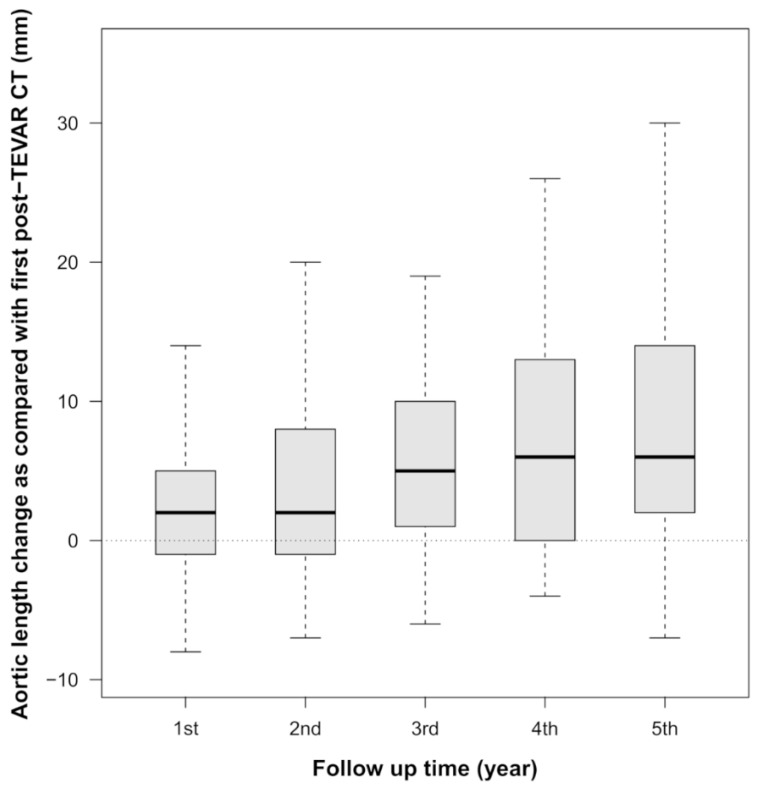
Aortic length change after Thoracic endovascular repair (TEVAR). The median change in aortic length from innominate artery to celiac artery (INA–CA) between the immediate follow-up CT and the first, second, third, fourth, and fifth years’ follow-up CT, was 3, 4, 6, 7, and 7 mm, respectively.

Regarding the length change, the median value of length change between the fifth annual follow-up CT and the immediate follow-up CT was an increase of 7 mm (IQR, 13) for INA–CA (Table 3, Figure 2), and an increase of 4 mm (IQR, 6) for STJ–INA, respectively. 

### 3.2. Elongation

The mean INA–CA length growth rate was 1.7 mm per year. Using the predefined elongation as a length of growth of 10 mm or more, 16 of 41 (39.0%) patients were classified as having elongation for INA–CA, while eight of 41 (19.5%) patients showed elongation for STJ–INA. The percentage of patients showing elongation increased during the follow-up, with the percentage of patients showing elongation at INA–CA being 12%, 22%, 32%, 32%, and 39% at the first, second, third, fourth, and fifth annual follow-ups. The proportion of patients with INA–CA elongation by the fifth year is larger than that by the first year (*p* < 0.01). The percentage of patients showing elongation at the STJ–INA segment during the follow-up was 2%, 5%, 12%, 15%, and 20% at the first, second, third, fourth, and fifth year follow-up (Table 4). 

Analysis for the association of patient, aortic, and procedural factors with elongation showed that hypertension and diabetes mellitus status were not associated with the proportion of elongation of the INA–CA aortic segment between group A and group B patients (*p* = 0.26 and 0.29, respectively). The age at TEVAR is older, and the maximal stent graft diameter, the maximal stent graft length, the pre-TEVAR native aortic STJ–INA, and the INA–CA length is larger, in group B patients (All *p* < 0.01) (Table 5).

## 4. Discussion

The current study showed that, at the follow-up CT, there was lengthening of the thoracic aorta, including the sinotubular junction to innominate artery and innominate artery to celiac artery, in patients receiving TEVAR.

Previous studies had found aortoiliac elongation after endovascular aortic aneurysm repair. Mean differences in aortic length were noted at multiple aortoiliac segments, while there was no significant change in iliofemoral segments. The changes were significant at three years after the procedure [14]. In a study about the remodeling of the aortic arch after hybrid TEVAR, Spinella et al. [15] showed that the aortic length along the centerline between the aortic root and celiac artery increased when comparing the 1 year follow-up CT and the 1 month follow-up CT; the subsegment analysis demonstrated that the aortic root to the proximal edge of the left subclavian artery showed significant elongation in the first year, while the left subclavian artery to the distal landing zone showed increased length, but was not statistically significant. Our study consisted a long-term follow-up of 5 years, and found that both the centerline length from the STJ to INA and the length from the INA to CA increased. This could be due to be that fact that the elongation was a progressive and ongoing process which was probably not statistically significant in the first year.

The mechanism of lengthening of the aorta was proposed to be due to the aging process, as the elastic and collagen component of the vascular structure likely degenerate and cause elongation [16]. Pulse pressure has been reported to be associated with the aortic root diameter with an inverse relation [17], and high pulse pressure predicted more rapid expansion in small abdominal aortic aneurysms [18]. The arteries could undergo active remodeling [19] and the geometry change in the arteries could impact the arterial function [20]. The arteries can have a vicious cycle between blood pressure elevation and arterial stiffening, which reflects active remodeling in the arteries’ response to hemodynamic stimuli [21].

The effect of lengthening the aorta impacts the following aspects. Riesterer et al. stated that elongation could cause migration, [22]. A study comparing patients with and without stent graft migration, found that there was more elongation of the aorta [11]. Migration of the stent graft after TEVAR could cause an incomplete seal, and may cause complications, such as endoleak. In a study following up patients with residual dissection after TEVAR, the relative movement of the stent graft along the aorta was minimal, at a median value of 2 (0–5 mm) [23]. 

Migration has been commonly defined as having a movement of more than 5 or 10 mm between the stent graft tip and an anatomical landmark along the aorta [13,24]. If there was elongation of the aortic segment distal to the stent graft and proximal to the distal landmark, such as the celiac artery, the stent graft could be misclassified as migrating cranially [12]. Knowing the elongation is essential for correctly determining stent migration. The current study showed that 39% of patients—a considerable proportion—receiving TEVAR showed elongation by the fifth year, therefore, surgeons or interventional radiologists should be cautious when identifying migration at long-term follow-up.

There are several limitations of the study. First, to know the longitudinal change in the aortic length, this study only included patients who received regular yearly follow-up; whether the result of individual timepoints could be applied to all patients receiving TEVAR is unknown. Second, non-ECG-gated CT scans were used for the measurements. While measurement ECG-gated CT scan images could be more accurate in the prospective study setting, the real-world daily practice of CT aortography is mainly performed with ECG-gated CT.

## 5. Conclusions

The current longitudinal study showed the aorta’s progressively lengthening after thoracic endovascular aortic repair. The mean lengthening rate from the innominate artery to the celiac artery was 1.7 mm per year.

## Figures and Tables

**Table 1:** Patient characteristics (*n* = 41).

Characteristics	Value ^1^	
Demographics		
Age, years	56	(19)
Sex		
Female	5	(12)
Male	36	(88)
Comorbidities		
Hypertension	33	(81)
Diabetes Mellitus	4	(10)
Coronary artery disease	3	(7)
Congestive heart failure	1	(2)
Chronic renal failure	1	(2)
Previous stroke	2	(5)
Hyperlipidemia	0	(0)
Collagen Vascular disease	1	(2)
Aortic characteristics		
Type		
Aortic dissection	21	(51)
Thoracic aortic aneurysm	15	(37)
Penetrated atherosclerotic ulcer	1	(2)
Intramural hematoma	3	(7)
Blunt aortic injury	1	(2)
Complicated presentation	17	(42)
TEVAR characteristics		
Stent graft number		
1	21	(51)
2	19	(46)
3	1	(2)
Stent graft type		
Stainless-steel based	33	(80)
Nitinol-based	8	(20)
Maximal stent graft diameter, mm	36	(4)
Maximal stent graft length, mm	194	(48)
Zone of repair		
1	10	(24)
2	16	(39)
3	15	(37)

^1^ Unless otherwise specified, the data are presented as No. (%) for categorical variables and as the median (interquartile range) for continuous variables. TEVAR: thoracic endovascular repair.

**Table 2:** Length of the aortic segment stratified by follow-up interval.

Time Interval	Aortic Segment ^1^							
	STJ–INA		INA–CA		LCCA–CA		LSCA–CA	
Pre-TEVAR	88	(14)	284	(34)	273	(31)	258	(22)
Immediate post-TEVAR	87	(11)	283	(30)	278	(30)	255	(25)
First year	88	(12)	285	(37)	276	(41)	260	(34)
Second year	88	(11)	289	(42)	279	(40)	264	(29)
Third year	89	(14)	289	(42)	281	(40)	264	(31)
Fourth year	90	(13)	291	(41)	281	(41)	263	(32)
Fifth year	91	(12)	294	(41)	283	(41)	263	(33)

^1^ The data are presented as median value (interquartile range), unit, mm. TEVAR: thoracic endovascular repair; STJ: sinotubular junction; INA: innominate artery; LCCA: left common carotid artery; LSCA: left subclavian artery; CA: celiac artery.

**Table 3:** Length difference of the aorta as compared with immediate post-TEVAR computed tomography (CT).

Time Interval	Aortic Segment ^1^							
	STJ–INA		INA–CA		LCCA–CA		LSCA–CA	
First year	0	(7)	3	(7)	2	(6)	0	(6)
Second year	2	(7)	4	(10)	2	(9)	2	(8)
Third year	1	(9)	6	(12)	5	(9)	5	(10)
Fourth year	3	(8)	7	(10)	6	(13)	4	(11)
Fifth year	4	(6)	7	(13)	6	(12)	4	(14)

^1^ The data are presented as median value (interquartile range), unit, mm. STJ: sinotubular junction; INA: innominate artery; LCCA: left common carotid artery; LSCA: left subclavian artery; CA: celiac artery.

**Table 4:** Percentage of patients showing elongation in aortic segment at the fifth year.

Segment	No. ^1^	(%)
STJ–INA	8	(20)
INA–CA	16	(39)
LCCA–CA	14	(34)
LSCA–CA	12	(29)

^1^ The data are presented as the number of patients showing lengthening of the aortic segment of 10 mm or more, which was classified as having elongation. STJ: sinotubular junction; INA: innominate artery; LCCA: left common carotid artery; LSCA: left subclavian artery; CA: celiac artery.

**Table 5:** Patient, aortic, procedural characteristics and elongation.

Factors ^1^	Group A ^2^ (*N* = 25)		Group B ^3^ (*N* = 16)		*p*-Value
Patient characteristics					
Gender					0.63
Female	4	(16)	1	(6)	
Male	21	(84)	15	(94)	
Age, years	55	(20)	60	(17)	<0.01
Comorbidities					
Hypertension					0.12
No	7	(28)	1	(6)	
Yes	18	(72)	15	(94)	
Diabetes mellitus					0.64
No	23	(92)	14	(88)	
Yes	2	(8)	2	(12)	
Coronary artery disease					0.55
No	24	(96)	14	(88)	
Yes	1	(4)	2	(12)	
Collagen vascular disease					1.00
No	24	(96)	16	(100)	
Yes	1	(4)	0	(0)	
Congestive heart failure					0.39
No	25	(100)	15	(94)	
Yes	0	(0)	1	(6)	
Aortic characteristics					
Complicated presentation					1.00
No	15	(60)	9	(56)	
Yes	10	(40)	7	(44)	
Innominate artery to celiac artery length	280	(38)	288	(26)	<0.01
Sinotubular junction to innominate artery	84	(12)	92	(14)	<0.01
TEVAR characteristics					
Repair zone					0.33
1	5	(20)	5	(31)	
2	12	(48)	4	(25)	
3	8	(32)	7	(44)	
Stent graft number					0.85
1	12	(48)	9	(56)	
2	12	(48)	7	(44)	
3	1	(4)	0	(0)	
Stent graft type					0.12
Nitinol-based	7	(28)	1	(6)	
Stainless steel-based	18	(72)	15	(94)	
Maximal stent graft diameter, mm	34	(2)	38	(6)	<0.01
Maximal stent graft length, mm	160	(48)	196	(46)	<0.01

^1^ Unless otherwise specified, the data are presented as no. (%) for categorical variables and as the median (interquartile range) for continuous variables. ^2^ Group A are patients showing a lengthening of innominate artery (INA)–celiac artery (CA) aortic segment of less than 10 mm between the fifth annual follow-up computed tomography (CT) and the immediate post-thoracic endovascular aortic repair (TEVAR) follow-up CT. ^3^ Group B are patients showing a lengthening of INA–CA aortic segment by 10 mm or more between the fifth annual follow-up CT and the immediate post-TEVAR follow-up CT.

## References

[1] Boufi M., Guivier-Curien C., Loundou A.D., Deplano V., Boiron O., Chaumoitre K., Gariboldi V., Alimi Y.S. (2017). Morphological analysis of healthy aortic arch. Eur. J. Vasc. Endovasc. Surg..

[2] Adriaans B.P., Heuts S., Gerretsen S., Cheriex E.C., Vos R., Natour E., Maessen J.G., Sardari Nia P., Crijns H., Wildberger J.E. (2018). Aortic elongation part I: The normal aortic ageing process. Heart.

[3] Kruger T., Forkavets O., Veseli K., Lausberg H., Vohringer L., Schneider W., Bamberg F., Schlensak C. (2016). Ascending aortic elongation and the risk of dissection. Eur. J. Cardiothorac. Surg..

[4] Heuts S., Adriaans B.P., Gerretsen S., Natour E., Vos R., Cheriex E.C., Crijns H., Wildberger J.E., Maessen J.G., Schalla S. (2018). Aortic elongation part II: The risk of acute type A aortic dissection. Heart.

[5] Grigorian A., Spencer D., Donayre C., Nahmias J., Schubl S., Gabriel V., Barrios C. (2018). National trends of thoracic endovascular aortic repair versus open repair in blunt thoracic aortic injury. Ann. Vasc. Surg..

[6] Yamaguchi T., Nakai M., Sumita Y., Nishimura K., Tazaki J., Kyuragi R., Kinoshita Y., Miyamoto T., Sakata Y., Nozato T. (2019). Editor’s choice–Endovascular repair versus surgical repair for Japanese patients with ruptured thoracic and abdominal aortic aneurysms: A nationwide study. Eur. J. Vasc. Endovasc. Surg..

[7] Thrumurthy S.G., Karthikesalingam A., Patterson B.O., Holt P.J., Hinchliffe R.J., Loftus I.M., Thompson M.M. (2011). A systematic review of mid-term outcomes of thoracic endovascular repair (TEVAR) of chronic type B aortic dissection. Eur. J. Vasc. Endovasc. Surg..

[8] Daye D., Walker T.G. (2018). Complications of endovascular aneurysm repair of the thoracic and abdominal aorta: Evaluation and management. Cardiovasc. Diagn. Ther..

[9] Fairman A.S., Beck A.W., Malas M.B., Goodney P.P., Osborne N.H., Schermerhorn M.L., Wang G.J. (2020). Reinterventions in the modern era of thoracic endovascular aortic repair. J. Vasc. Surg..

[10] O’Neill S., Greenberg R.K., Resch T., Bathurst S., Fleming D., Kashyap V., Lyden S.P., Clair D. (2006). An evaluation of centerline of flow measurement techniques to assess migration after thoracic endovascular aneurysm repair. J. Vasc. Surg..

[11] Geisbusch P., Skrypnik D., Ante M., Trojan M., Bruckner T., Rengier F., Bockler D. (2019). Endograft migration after thoracic endovascular aortic repair. J. Vasc. Surg..

[12] Alberta H.B., Takayama T., Panthofer A., Cambria R.P., Farber M.A., Jordan W.D., Matsumura J.S. (2018). Thoracic endovascular aortic repair migration and aortic elongation differentiated using dual reference point analysis. J. Vasc. Surg..

[13] Fillinger M.F., Greenberg R.K., McKinsey J.F., Chaikof E.L., Society for Vascular Surgery Ad Hoc Committee on TEVAR Reporting Standards (2010). Reporting standards for thoracic endovascular aortic repair (TEVAR). J. Vasc. Surg..

[14] Chandra V., Rouer M., Garg T., Fleischmann D., Mell M. (2015). Aortoiliac elongation after endovascular aortic aneurysm repair. Ann. Vasc. Surg..

[15] Spinella G., Finotello A., Conti M., Faggiano E., Gazzola V., Auricchio F., Chakfe N., Palombo D., Pane B. (2019). Assessment of geometrical remodelling of the aortic arch after hybrid treatment. Eur. J. Cardiothorac. Surg..

[16] Ohyama Y., Redheuil A., Kachenoura N., Ambale Venkatesh B., Lima J.A.C. (2018). Imaging insights on the aorta in aging. Circ. Cardiovasc. Imaging.

[17] Farasat S.M., Morrell C.H., Scuteri A., Ting C.T., Yin F.C., Spurgeon H.A., Chen C.H., Lakatta E.G., Najjar S.S. (2008). Pulse pressure is inversely related to aortic root diameter implications for the pathogenesis of systolic hypertension. Hypertension.

[18] Cronenwett J.L., Sargent S.K., Wall M.H., Hawkes M.L., Freeman D.H., Dain B.J., Cure J.K., Walsh D.B., Zwolak R.M., McDaniel M.D. (1990). Variables that affect the expansion rate and outcome of small abdominal aortic aneurysms. J. Vasc. Surg..

[19] Gibbons G.H., Dzau V.J. (1994). The emerging concept of vascular remodeling. N. Engl. J. Med..

[20] Scuteri A., Chen C.H., Yin F.C., Chih-Tai T., Spurgeon H.A., Lakatta E.G. (2001). Functional correlates of central arterial geometric phenotypes. Hypertension.

[21] Scuteri A., Morrell C.H., Orru M., AlGhatrif M., Saba P.S., Terracciano A., Ferreli L.A., Loi F., Marongiu M., Pilia M.G. (2016). Gender specific profiles of white coat and masked hypertension impacts on arterial structure and function in the SardiNIA study. Int. J. Cardiol..

[22] Riesterer T., Beyersdorf F., Scheumann J., Berezowski M., Schrofel H., Kondov S., Czerny M., Rylski B. (2019). Accuracy of deployment of the Relay non-bare stent graft in the aortic arch. Interact. Cardiovasc. Thorac. Surg..

[23] Lescan M., Czerny M., Berezowski M., Andic M., Bamberg F., Beyersdorf F., Schlensak C., Rylski B. (2019). Morphologic performance analysis of the Relay nonbare stent graft in dissected thoracic aorta. J. Vasc. Surg..

[24] Canaud L., Marty-Ane C., Ziza V., Branchereau P., Alric P. (2015). Minimum 10-year follow-up of endovascular repair for acute traumatic transection of the thoracic aorta. J. Thorac. Cardiovasc. Surg..

